# Gene Therapy for *LMNA*-related Congenital Muscular Dystrophy (L-CMD) by Trans-Splicing

**DOI:** 10.1186/1750-1172-10-S2-O11

**Published:** 2015-11-11

**Authors:** Feriel Azibani, Anne T Bertrand

**Affiliations:** 1 Sorbonne Universités, UPMC Univ Paris 06, INSERM UMRS974, CNRS FRE3617, Center for Research in Myology, Paris, France

## 

*LMNA*-related Congenital Muscular Dystrophy (L-CMD) is a rare genetic disorder characterized by the onset of selective axial weakness and wasting in the first year of life with limited motor achievements, associated with multiple severe contractures and frequent respiratory failure requiring early ventilatory support. We identified heterozygous *de novo* mutations in *LMNA*, encoding lamins A/C, as responsible for this sub-group of CMD in which no therapeutic treatment is available[1]. Lamins A/C are nuclear envelope proteins, ubiquitously expressed in all post mitotic cells, which play essential roles in the nucleus structure and in the regulation of gene expression. We generated the first Knock-In mouse model of L-CMD (KI-*Lmna^delK32^*) reproducing a *LMNA* mutation identified in L-CMD patients. Homozygous mice die within the first 3 weeks of life from striated muscles maturation delay and severe metabolic defects[2]. Heterozygous mice develop an isolated dilated cardiomyopathy and die by one year of age[3]. We aim to assess the possibility of LMNA-mRNA repair by spliceosome-mediated RNA trans-splicing (SMarT) as a potential therapeutic approach for L-CMD. This gene therapy strategy will allow inhibition of mutated LMNA transcript expression for the benefit of corresponding wild type transcripts. We developed 5'-RNA pre-trans-splicing molecules (PTM) capable of repairing the murine LMNA transcripts. Efficiency of these PTM was assessed *in vitro* in C2C12 cells and *in vivo* using Adeno-Associated Virus (AAV) transduction in *tibialis anterior* of WT mice. We will now determine the ability of the best PTM to restore normal muscular phenotype, *in vitro* in KI myoblasts/myotubes and *in vivo* after injection of AAV-PTM vectors in new born homozygous and adult heterozygous mice. Histological and metabolic parameters will be monitored to evaluate the degree of phenotype rescue.
